# Mapping of Repetitive Sequences in *Brachyhypopomus brevirostris* (Hypopomidae, Gymnotiformes) from the Brazilian Amazon

**DOI:** 10.3390/ani14121726

**Published:** 2024-06-07

**Authors:** Paula Pinto Rodrigues, Milla de Andrade Machado, Ananda Marques Pety, Willam Oliveira da Silva, Julio Cesar Pieczarka, Cleusa Yoshiko Nagamachi

**Affiliations:** Laboratório de Citogenética, Centro de Estudos Avançados da Biodiversidade, Instituto de Ciências Biológicas, Universidade Federal do Pará, Belém 66075-750, Brazil; paula.rodrigues4.pr@gmail.com (P.P.R.); millaamachado@gmail.com (M.d.A.M.); anandapety3@gmail.com (A.M.P.); willam_oliveira@hotmail.com (W.O.d.S.); juliopieczarka@gmail.com (J.C.P.)

**Keywords:** repetitive sequences, FISH, Amazon Basin

## Abstract

**Simple Summary:**

Neotropical electric fish have a large diversity in the Amazon region. We investigated the karyotype of the species *Brachyhypopomus brevirostris* from two localities in Brazil’s northern region, Santarém in Pará state and Tefé in Amazonas state, using classical and molecular cytogenetics. Specimens from both localities presented the same karyotype. These are the first results regarding the distribution of repetitive sequences for *B. brevirostris* samples from the Tefé locality, and the first karyotypic description for the Santarém locality. These results differ from those previously described for samples from Humaitá (Amazon state). This karyotypic difference suggests that the Humaitá sample belongs to another species, which is reinforced in the recent redescription of the genus *Brachyhypopomus*.

**Abstract:**

*Brachyhypopomus* (Hypopomidae, Gymnotiformes) is a monophyletic genus consisting of 28 formally described species. Karyotypic data are available for 12 species. The same karyotype is described for two species (*B. brevirostris* and *B. hamiltoni*), as well as different karyotypes for the same species from distinct locations (*B. brevirostris*). In this context, *B. brevirostris* may constitute a cryptic species complex. Thus, in the present study, we analyzed the karyotype of *B. brevirostris*, from Santarém, Pará, and Tefé, Amazonas, using classical cytogenetics (conventional staining and C-banding) and molecular techniques (fluorescence in situ hybridization using 18S rDNA, 5S rDNA, U2 snRNA, and telomeric probes). The results show that samples from both locations present 2n = 38, with all chromosomes being acrocentric (FC = 38a). In both populations, 18S rDNA sequences are present on only one pair of homologous chromosomes and telomeric sequences occur only at the ends of the chromosomes. In the Tefé sample, the 5S rDNA occurs in two pairs, and the U2 snRNA in three pairs. These results are the first descriptions of these sequences for *B. brevirostris* samples from the Tefé locality, as well as the first karyotypic description for the Santarém locality. Future cytotaxonomic studies of this genus can benefit from these results.

## 1. Introduction

South America’s hydrological landscape is shaped by a rich network of hydrobasins. Many of these hydrobasins sustain diverse ecosystems and provide crucial resources to both humans and wildlife, such as the Amazon, Orinoco, Paraná, and many others. One of the largest and most significant hydrobasins in the world, the Amazon Basin, is known for its unparalleled biodiversity and Amazon River [1].

*Brachyhypopomus* Mago-Leccia 1994, is one of the six genera of electric fish in the family Hypopomidae (Gymnotiformes), widely distributed in the Neotropical region and inhabiting the hydrobasins of South America. It occurs from the La Plata River, in Argentina (35° S), to the Tuira River, in Panama (8° N), and can be found in all South American countries except Chile. It presents greater diversity and abundance in the Amazon Basin [2,3,4,5], which is considered to be the center of origin of this genus, from which it would have dispersed to adjacent basins [5,6].

*Brachyhypopomus* species, as well as other Gymnotiformes, can generate and detect electrical potentials in water through an electrical organ (EOD) and specialized sensory cells that are organized throughout the animal’s body, called electroreceptors, which are responsible for electrocommunication and active electrolocation [7,8,9,10,11]. These species are cryptically pigmented, nocturnal predators of small and medium-sized aquatic invertebrates and can occur in slow-flowing and shallow habitats, as well as streams, swamps, and seasonal floodplains, and can be an abundant component of the local ichthyofauna [5,12]. *Brachyhypopomus* is monophyletic, as confirmed by parsimony and Bayesian total evidence-based phylogenetic analyses [5], with 28 valid species (Supplementary Table S1), of which 15 have been recently described [6]. The first species described for the genus was *B. brevirostris* by Staindachener in 1868 (as *Rhamphichthys brevirostris*), which is widely distributed in the Amazon, Orinoco, and Guiana basins [6].

Karyotypic data were described for 12 of the 28 species [13,14,15,16,17,18] (Table 1). Different species of *Brachyhypopomus* differ in 2n (26 to 44), KF, and the number of chromosomes carrying NOR, and may be simple or multiple (Table 1). There are also karyotypes with the multiple sex system X_1_X_1_X_2_X_2_/X_1_X_2_Y found in three species: *B. pinnicaudatus* (2n = 41/42) and *B. flavipomus* (2n = 43/44), both from Mamirauá—AM [15], and *B. gauderio* (2n = 41/42) from the Tietê River—SP [16], from Porto Rico—PR [17], and the Paranapanema River—PR [18], all from the Upper Paraná River. *Brachyhypopomus brevirostris*, from the Madeira River in Humaitá—AM, was the first species of the genus to have its karyotype studied, showing a diploid number (2n) of 36 chromosomes and a karyotypic formula (FC) of 6m/sm + 30st/a [13]. This 2n is shared by the species *B. hamiltoni* from Tefé—AM [14], whose karyotype also shares the same FC. On the other hand, *B. brevirostris* from the Solimões River in the Tefé—AM region presents a karyotype with 2n = 38 and FC = 38st/a [14], which is different from that found in Humaitá.

Cytogenetics has become an important tool for detecting biodiversity [19,20,21], revealing a large amount of information about evolutionary processes within a group, such as chromosomal rearrangements, structural and/or numerical polymorphisms, sexual chromosome systems, and variations associated with the geographic distribution of some species and/or populations [20,21,22].

Although classical cytogenetics has allowed good insights into understanding chromosomal diversity and evolutionary processes, in fish, access to the genome was limited, a fact that made it difficult to detect different levels of genetic divergence [21,23]. The emergence of molecular cytogenetics using fluorescence in situ hybridization (FISH) has resulted in a more precise resolution of the physical location of chromosomes [24].

The aim of using the FISH technique is to understand the structural nature of chromosomes [25], trace the origin and evolution of sex chromosomes [26] and their behavior in the cells’ meiotic process [27], resolve taxonomic questions, and even provide information on the evolution of DNA sequences [28,29,30]. Chromosomal DNA mapping by FISH has been an indispensable tool in understanding chromosomal dynamics and evolution, providing a more refined way of researching chromosomal differentiation. The study of repetitive sequences is therefore crucial to understanding their dynamics and the evolution of the genome, as well as to identifying possible genetic markers for mapping the location of these sequences and indicating their conservation or diversity. Intending to expand our knowledge about the chromosomal structure and the dynamics of repetitive DNA sequences in the *Brachyhypopomus* genome, we present, for the first time, the karyotype and chromosomal location of three repetitive DNA classes (18S, 5S rDNA, and U2 snDNA) in *B. brevirostris* from Santarém, Pará, and the Tefé, Amazonas, in the Amazon Basin.

## 2. Materials and Methods

### 2.1. Sampling

**Figure 1:**
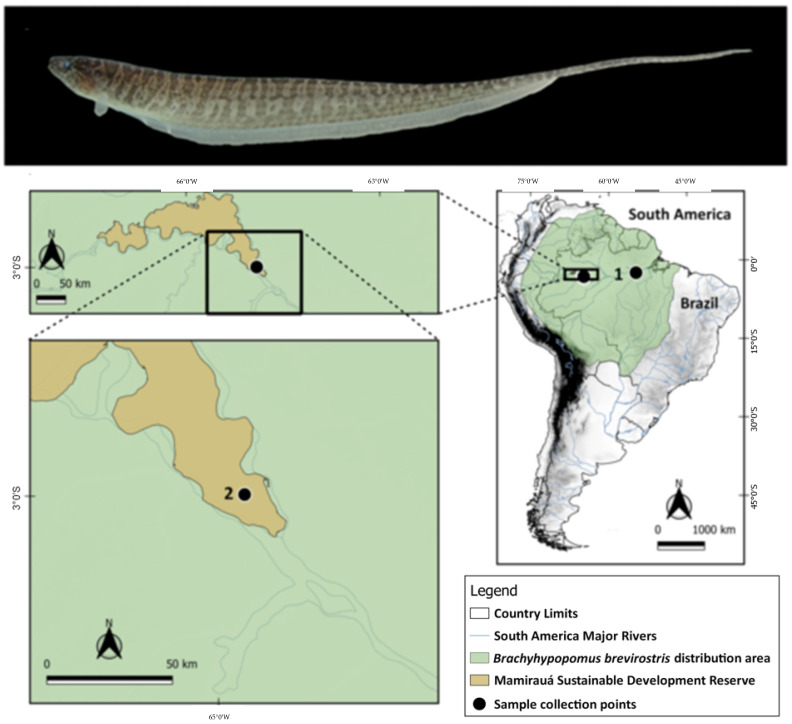
Photo of a specimen of *Brachyhypopomus brevirostris*; map showing collection point referring to the *B. brevirostris* samples from this work. 1: Aramanaí stream—Santarém; 2: Mamirauá Reserve—Tefé. The map was made using QGIS v. 3.10.7. The shapefiles containing country boundaries, elevation, and hydrography were obtained from DIVA-GIS [32], at the link https://www.diva-gis.org/gdata accessed on 1 February 2024.

Samples of *B. brevirostris* were obtained from two locations: the Mamirauá Reserve, in the region of Tefé—AM, and the municipality of Santarém—PA, from the Aramanaí River (Table 2, Figure 1). The specimens were located and collected with the aid of an electric discharge detector and nylon nets, in addition to the use of flashlights to better visualize the environment. The sample collections took place from dusk, as species in this order have nocturnal habits, being more frequently located on riverbanks. All specimens were processed in the field and euthanized with eugenol [31].

### 2.2. Cytogenetic Analysis

Metaphase chromosomes were obtained by direct extraction from the head kidney [33] after inducing mitosis by fermentation [34]. C-Banding [35], fluorescence in situ hybridization (FISH) with 18S rDNA [36], 5S rDNA [37], U2 snRNA [38], and telomeric sequence (TTAGGG)n [39] probes followed the protocol previously described [40], using the following primers: 18Sf (5′-CCG CTT TGG TGA CTC TTG AT-3′), 18Sr (5′-CCG AGG ACC TCA CTA AAC CA-3′) [36], 5Sf (5′-GCCACACCACCCTGAACAC-3′), 5Sr (5′-GCCTACGACACCTGGTATTC-3′) [37], U2f (3′-TCTCGGCCTATATTGGCTAA-5′) and U2r (3′-GACGGTAGCTGCAATACCGG-5′) [38]. The 18S rDNA amplification cycles comprised a denaturation for 5 min at 95 °C; 30 cycles of 1 min at 95 °C, 30 seg. at 50 °C, and 45 seg. at 72 °C; a final extension of 5 min at 72 °C; and a cooling period at 4 °C. The 5S rDNA amplification cycles comprised a denaturation for 4 min at 95 °C; 35 cycles of 1 min at 95 °C, 1 min at 60 °C, and 2 min at 74 °C; a final extension of 5 min at 74 °C; and a cooling period at 4 °C. The U2 snRNA amplification cycles comprised a denaturation for 4 min at 95 °C; 30 cycles of 1 min at 95 °C, 1 min at 60 °C, and 2 min at 74 °C; a final extension of 5 min at 74 °C; and a cooling period at 4 °C. The primers (TTAGGG)n and (CCCTAA)n [39] were used to obtain telomeric sequences. PCR was performed with the following profile: 5 min at 94 °C; 35 cycles of 1 min at 94 °C; 30 seg. at 60 °C; 1.5 min at 72 °C, and 5 min at 72 °C. The probes were labeled by PCR using the incorporation of biotinylated dUTP (Invitrogen), or by nick-translation with the BioNick Labeling System kit (Invitrogen, Waltham, MA, USA) for labeling with biotin, and Dig-nick (Roche, Mannheim, Germany) for labeling with digoxigenin. Labeling signals were detected with avidin-Cy3 or anti-digoxigenin-FITC, in addition to DAPI for staining. Images were observed andcaptured using a Zeiss Imager D2 photomicroscope (Zeiss, Oberkochen, Germany) and images were acquired with an Axiocam 503 camera (Zeiss, Oberkochen, Germany) and processed using the ZEN software (Zeiss, Oberkochen, Germany, version 2.0.0.0). The karyotypes were organized using Photoshop CC 2024 (Adobe Systems, San Jose, CA, USA, version 25.10.0). Chromosomes were classified into two groups: metacentric/submetacentric (m/sm) and subtelocentric/acrocentric (st/a) and arranged in decreasing order of size in each group.

## 3. Results

**Figure 2:**
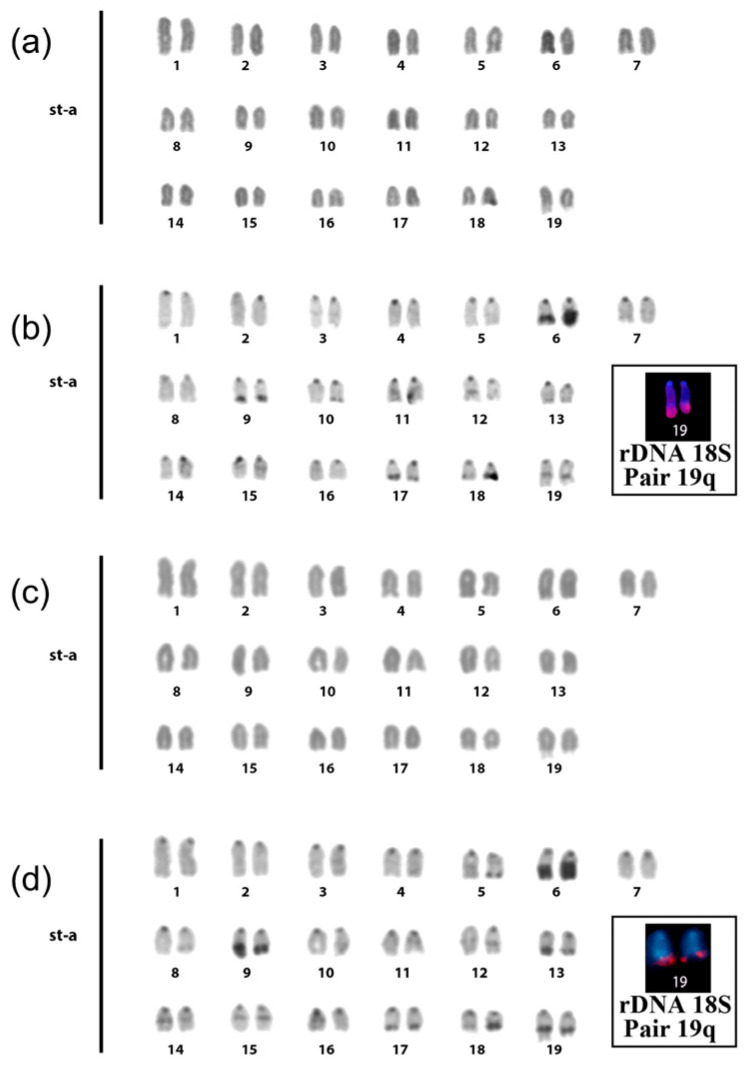
Karyotype of *Brachyhypopomus brevirostris*: (**a**) conventional staining of the sample from Santarém—PA; (**b**) C-Banding, from the Santarém—PA sample; (**c**) conventional staining of the sample from the Mamirauá Reserve, Tefé—AM region; (**d**) C-banding of the sample from the Mamirauá Reserve, Tefé—AM region.

The diploid number (2n) of *Brachyhypopomus brevirostris* specimens from both locations is equal to 38, with all chromosomes being acrocentric (Figure 2a,c).

Constitutive heterochromatin (HC) is distributed in small blocks found in the centromeric region of all chromosomes, with some pairs showing a small interstitial band on the long arm and others revealing a small distal band on the long arm. A larger, heteromorphic heterochromatic distal band was seen in the long arm of one of the pair 6 homologs (Figure 2b,d).

**Figure 3:**
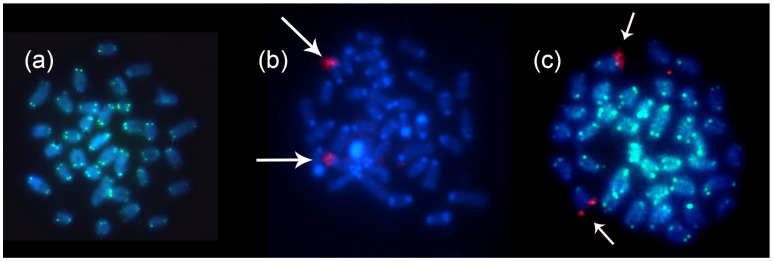
FISH with 18S rDNA and telomeric probes, without evidence of ITS. (**a**) FISH with Telomeric probe, sample from Santarém—PA. (**b**) FISH with 18S rDNA probe (red) indicated by white arrows, hybridizing to a chromosomal pair (19q), sample from Santarém–PA. (**c**) Double FISH with 18S rDNA probe (red) indicated by white arrows, hybridizing to a chromosomal pair (19q) and telomeric probe (green), sample from the Mamirauá Reserve, Tefé–AM region.

Fluorescence in situ hybridization (FISH) with a telomeric sequence probe showed a signal in the terminal region of all chromosomes, with no interstitial marking observed (Figure 3a,c, in green). FISH with 18S rDNA probes showed simple signals in the distal region of the long arm of chromosome pair 19 (Figure 3b,c, in red shown by arrows).

**Figure 4:**
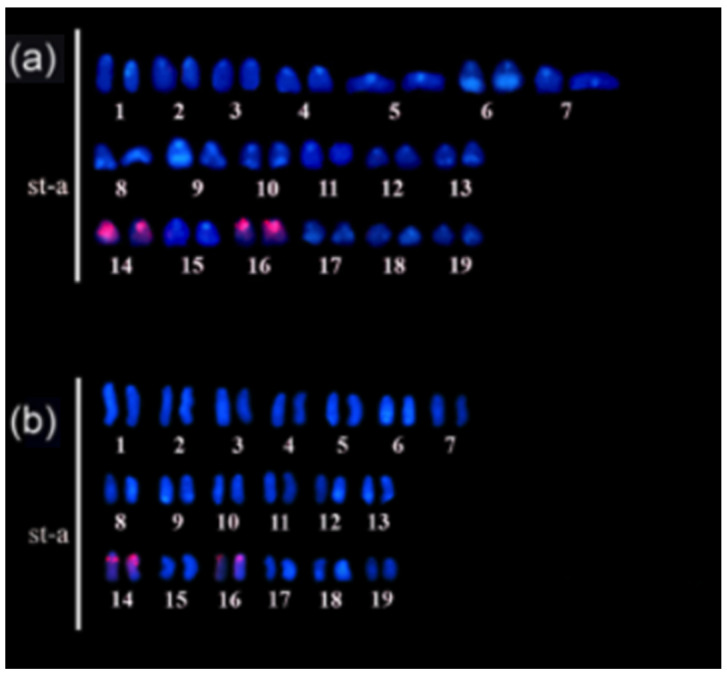
FISH with 5S rDNA probes hybridizing to two chromosomal pairs: (**a**) sample from Tefé—AM; (**b**) sample from Santarém—PA.

**Figure 5:**
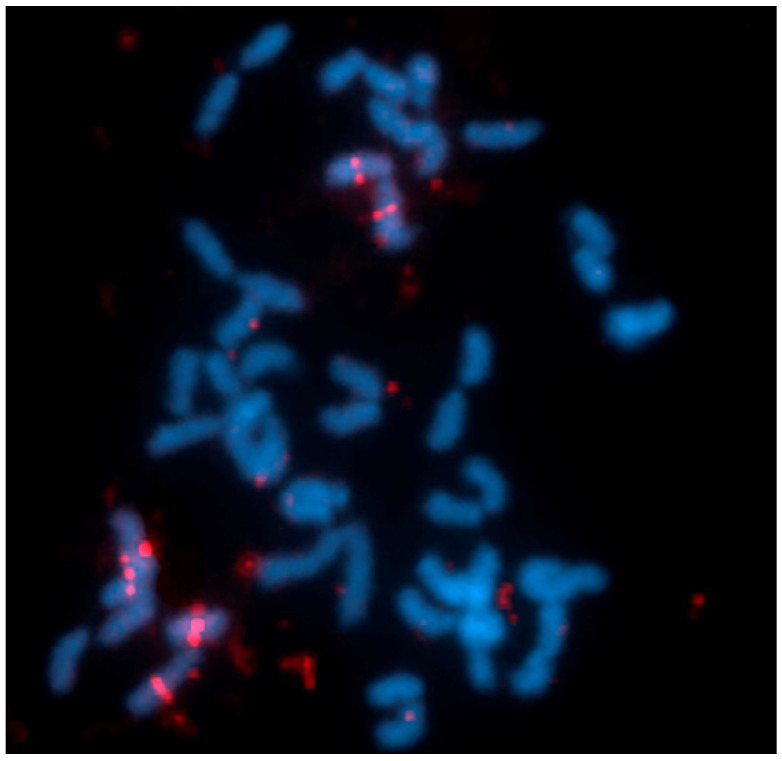
FISH with sn-U2 probe (red), signal in multiple chromosomes of *B. brevirostris*.

FISH results with 5S rDNA show marking of 5S rDNA in pairs 14 and 16 (Figure 4), and of U2 snRNA sequences (obtained from samples of Tefé—AM region) in multiple chromosomes; it was not possible to identify the pairs in the karyotype (Figure 5).

## 4. Discussion

The 2n = 38 found in *B. brevirostris* is within the variation found for the superfamily Rhamphichthyoidea, which varies from 26 chromosomes for *B*. cf. *draco* [18], to 50 for *Hypopygus lepturus* [13], *Steatogenys duidae*, and *Steatogenys elegans* [41]. Using ChromEvol, (version 2.0), a software package that implements a series of likelihood models regarding the pathways by which the evolution of chromosome number proceeds, Takagui et al. [18] estimated that 2n = 34 is the ancestral condition for this clade, just as in *B. beebei* and *B. hamiltoni*, which have 40 and 36 chromosomes, respectively. *B. brevirostris* also had its karyotype originated by centromeric fissions.

The karyotype of *B. brevirostris* in the present study is similar to that found for the Tefé—AM region [14], presenting the same 2n, KF, and constitutive heterochromatin distribution pattern, with the same block size heteromorphism in pair 6. This size heteromorphism, due to the difference in the size of the heterochromatic block, can be explained by a constitutive heterochromatin amplification mechanism between the pairs. This characteristic added to a set of data for this species can be used as a cytogenetic marker, as has been suggested for other neotropical fish species [42,43,44].

*Brachyhypopomus brevirostris* from the present study, despite sharing the diploid number with *B. herdersoni* and *B. regani*, differs in its karyotypic formulas (Table 1), which result from events that modify chromosomal morphology, but do not alter 2n, such as pericentric inversions, translocations of chromosomal segments, and repositioning of the centromere [14,45].

Positive C-band regions are coincident with positive DAPI staining, suggesting that constitutive heterochromatin has a DNA composition rich in A-T nucleotides [16,43]. Previous studies on the location of the Nucleolus Organizer Region (NOR) in Hypopomidae are only available for two genera. NOR presents a multiple system in *Brachyhypopomus gauderio* [17,18] and in *Microsternarchus bilineatus* from Rio Negro—AM [46], and a simple system in *Brachyhypopomus* cf. *draco* [18] and *Microsternarchus* aff. *bilineatus* from Santarém—PA [47]. It is possible to notice that there is a size heteromorphism present between the chromosomes of the NOR pair, a characteristic that is considered common, possibly due to tandem duplication, unequal crossing over between repetitive sequences, or accidental duplication [48]. Despite the difference between the NORs found, it is still not possible to establish a pattern of NOR distribution for this family, as there are little karyotypic data available for the genera of Hypopomidae.

Among the Hypopomidae, published data on 5S DNA and U2 snRNA are scarce or non-existent. *Microsternarchus bilineatus* from Rio Negro—AM presents 5S DNA signals in a single pair [46], different from *B. brevisrostris* in this study, which presented signals in two chromosome pairs. In the literature, there are no results of 5S rDNA and U2 snRNA sequences for *Brachyhypopomus* species, with the data from this study being the first to be presented. The U2 snRNA was previously studied in some Gymnotiformes genera [49,50,51,52], showing simple or multiple hybridization (Table 3), and, in some cases, the U2 snRNA is associated with the 5S rDNA, like in *Eigenmannia limbata*, *E. microstoma* [49], and *Eigenmannia* aff. *Trilineata* [50]. In this study, we found no association between 5S rDNA and U2 snRNA. We found multiple labeling for the U2 snRNA, and although we were unable to identify which pairs corresponded in the karyotype, the number of chromosome pairs with signals is similar to those of *Eigenmannia limbata* and *Archolaemus janeae*.

FISH using telomeric sequence probes showed no interstitial signals, which may suggest that chromosomal rearrangements that occurred during the evolution of the karyotype did not include the presence of these sequences or that they were modified after a fusion event [43,53].

**Figure 6:**
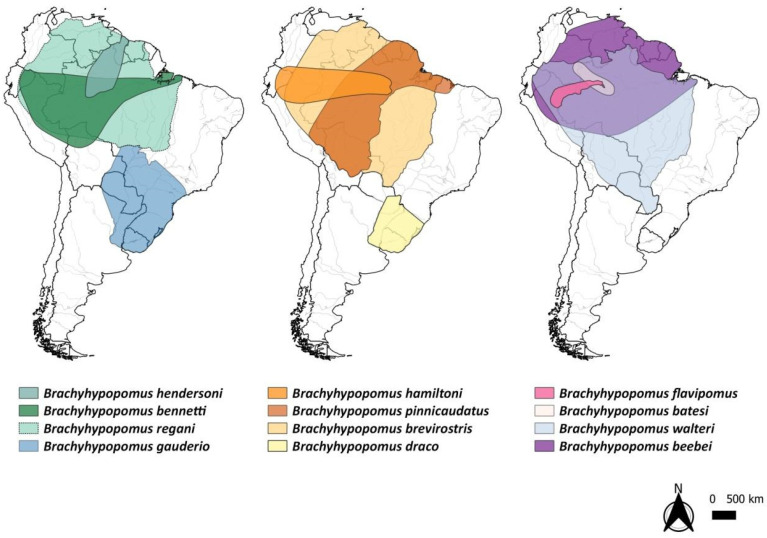
Maps showing the distribution areas of species of the genus *Brachyhypopomus* [6] that have cytogenetic data available in the literature. The map was made using QGIS v. 3.10.7. The shapefiles containing country boundaries, elevation, and hydrography were obtained from DIVA-GIS [32], at the link https://www.diva-gis.org/gdata, accessed on 1 February 2024.

*Brachyhypopomus brevirostris* is widely distributed in the northern portion of South America (Figure 6), occurring in various habitats and co-occurring geographically with 19 other congenera [6]. Of these, 12 have cytogenetic studies available in the literature, including *B. brevirostris* [14,15,16,17,18]. Most species of the genus *Brachyhypopomus* studied cytogenetically come from the Tefé region, located in the Amazon Basin, except *B. gauderio*, from the Upper Paraná River Basin and *B. draco* from the Tramandaí Basin in Rio Grande do Sul (Figure 6).

The karyotype of *B. brevirostris* in the present study (2n = 38, FC = 38st/a) differs from that described (2n = 36, FC = 6m/sm + 30st/a) for Humaitá [13], both in 2n and in the morphology of chromosomes (Table 1). Fusion/fission rearrangements explain the difference in 2n, and inversions and translocations can lead to changes in chromosome morphology. These karyotypic differences between specimens from distinct locations (Table 1; Figure 1) (Tefé—AM, samples from the present study, and Humaitá—AM for the literature sample [13]) may characterize different species and may be cryptic. The three sampled points, Humaitá (1), Tefé (2 to 6), and Santarém (7), of *Brachyhypopomus brevirostris*, form a triangle on the map (Figure 1), with the Madeira, Tefé, and Tapajós rivers of the three sampling points, respectively, having their mouths on the Amazon River. When the karyotype from Humaitá was published [13], it was assigned to the species *B. brevirostris*. At that time, only six species were described for the genus *Brachyhypopomus*. Currently, 15 species are described for this genus [6]. This recent study demonstrated that some previous *Brachyhypopomus* taxa were composed of more than one species [6]. Regarding the geographic distribution of the *Brachyhypopomus* species shown on the map (Figure 6), we can see a trend towards some specifically eurytopic species, which are more tolerant to a variety of environments and conditions. For example, *B. brevirostris, B. regain, B. hamiltoni, B. beebei*, and *B. walteri* occupy wider geographic areas in the Amazon region, than stenotopic species, except for *B. hamiltoni*. This has already been observed for other gymnotiform species, such as *Gymnotus carapo* and *Sternopygus macrurus*, as well as for other neotropical fish taxa [6,54]. Thus, the sample from Humaitá [13] may belong to another species of this genus, such as *B. hamiltoni* [14], which has the same karyotype (Table 1).

In addition to providing valuable insights regarding the overall species diversity in South American hydrobasins, *Brachyhypopomus* distribution can be used to identify biodiversity hotspots and areas that require priority conservation efforts [55]. We observed that sympatry is widespread in this genus (Figure 6), with overlap distribution occurring between three and eleven species, as *B. walteri* occurs in sympatry with *B. draco* and *B. gauderio*, while most other taxa have greater contact with more species and are more widespread. Even though *B. flavipomus* and *B. batesi* have smaller distribution areas, they are still sympatric with the other eight species.

**Figure 7:**
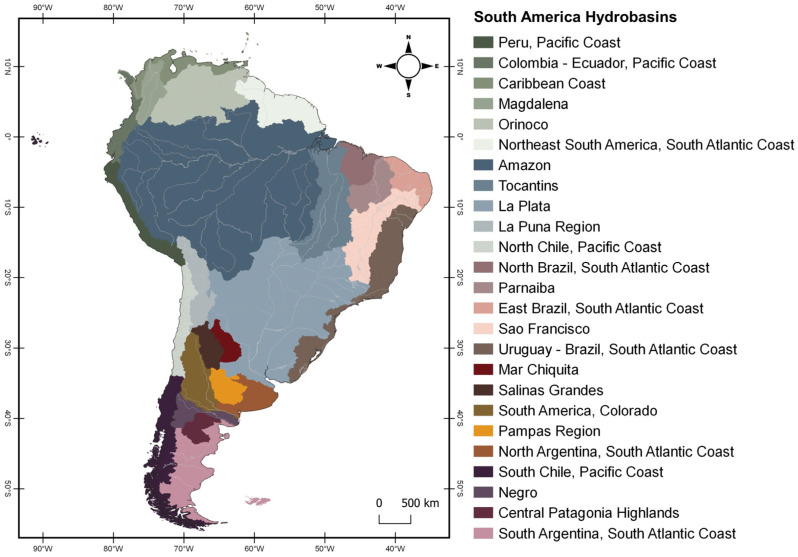
Map showing South America hydrobasins. The map was made using QGIS v. 3.10.7. The shapefiles containing country boundaries, elevation, and hydrography were obtained from DIVA-GIS [32], at the link https://www.diva-gis.org/gdata, accessed on 1 February 2024. Hydrobasins’ limits were based on Boschman et al., 2023 [58].

Furthermore, *Brachyhypopomus* can be used as a bioindicator species for the health of hydrobasin ecosystems by demonstrating the presence of suitable habitats and environmental conditions in hydrobasins [56]. *Brachyhypopomus* occurs in 8 out of the 25 hydrobasins of South America (Figure 7), and its distribution could be influenced by a variety of factors, including the quality of the water, its depth, and the availability of food and shelter [57]. *B. hamiltoni*, *B. flavipomus*, and *B. batesi* are endemic to the Amazon Basin; *B. gauderio* and *B. draco* occur at La Plata and Uruguay; *B. hendersoni* occurs in the Amazon and Northeast South America; *B. bennetti* is mostly distributed in the Amazon, north of Tocantins, and in a small portion of North Brazil; *B. regani* is widely spread in the Amazon, Orinoco, Northeast South America and Tocantins, and a small portion of North Brazil; *B. pinnicaudatus* occurs in the Amazon, Northeast South America, and small areas of the Tocantins and North Brazil; *B. brevirostris* occurs in the Amazon, Orinoco, Northeast South America, Tocantins, La Plata, and a small portion of North Brazil; *B. walteri* occurs in the Amazon, La Plata, Tocantins, and a small area of Northeast South America; and *B. beebei* occurs in the Amazon, Orinoco, Northeast South America, and small areas of Caribbean Coast.

As stated by [1], in South America, river configurations over millions of years have facilitated species dispersal, which has led to an increase in fish diversity. The species richness of Western Amazonia is extremely high and decreases from west to east [1], which is consistent with the pattern observed in *Brachyhypopomus*, whose majority of representatives reside in the Amazon, and *B. hendersoni*, *B. hamiltoni*, *B. flavipomus*, and *B. batesi* are found only in Western Amazon.

## 5. Conclusions

These results are the first descriptions of 18S rDNA, 5S rDNA, and U2 snRNA sequences for *B. brevirostris* samples from the Tefé locality, and the first karyotypic description for the Santarém locality. The karyotype described for *B. brevirostris* from Humaitá—AM is similar to that recently described for *B. hamiltoni* (2n = 36; FC = 6m/sm + 30st/a), which suggests the possibility of them being the same species. The cytogenetic data obtained in this study for the two populations of *Brachyhypopomus brevirostris* indicate that, even isolated, they maintained the karyotype, with no evidence of recent rearrangements. These results contribute to the karyotypic knowledge of the Hypopomidae family, especially for the genus *Brachyhypopomus*. These results are extremely important and will be a relevant reference for future comparative studies.

## Figures and Tables

**Table 1:** Karyotypic data available for the genus *Brachyhypopomus*.

Species	Locality	2n	Sex	Sex System	KF	NOR/18S rDNA	5S rDNA	U2 snDNA	Reference
*B. brevirostris*	Humaitá—AM	36			6m/sm + 30st/a				[13]
	Tefé—AM	38			38st/a				[14]
		38			38st/a	19q	14p; 16p	3 pairs	Present study
	Santarém—PA	38			38st/a	19q	14p; 16p	3 pairs	Present study
*B. pinnicaudatus*	Mamirauá—AM	41	M	X_1_X_1_X_2_X_2_/ X_1_X_2_Y	1m/sm + 40st/a				[15]
		42	F		42st/a				
*B. flavipomus*	Mamirauá—AM	43	M	X_1_X_1_X_2_X_2_/ X_1_X_2_Y	1m/sm + 42st/a				[15]
		44	F		44st/a				
*B. batesi*	Tefé—AM	40			38m/sm + 2st/a				[14]
*B. hendersoni*	Tefé—AM	38			34m/sm + 4st/a				[14]
*B. regani*	Tefé—AM	38			14m/sm + 24st/a				[14]
*B. beebei*	Tefé—AM	40			8m/sm + 32st/a				[14]
*B. hamiltoni*	Tefé—AM	36			6m/sm + 30st/a				[14]
*B. bennetti*	Tefé—AM	40			2m/sm + 38st/a				[14]
*B. walteri*	Tefé—AM	40			2m/sm + 38st/a				[14]
*B.* cf. *draco*	Lagoa dos Quadros—RS	26			2m + 24a	13p			[18]
*B. gauderio*	Porto Rico—PR	41	M	X_1_X_1_X_2_X_2_/ X_1_X_2_Y	1m + 40a	8 signals			[17]
		42	F		42a				
	Tietê River—SP	41	M	X_1_X_1_X_2_X_2_/ X_1_X_2_Y	1m + 40a				[16]
		42	F		42a				
	Paranapanema River—PR	41	M	X_1_X_1_X_2_X_2_/ X_1_X_2_Y	1m + 40a	2p, 5p, 1q, 16q			[18]
		42	F		42a				

Legend: 2n—Diploid number, KF—Karyotypic formula, M—male, F—female; [13]: Almeida-Toledo, 1978; [14]: Cardoso et al., 2018; [15]: Cardoso et al., 2015; [17]: Mendes et al., 2012; [16]: Almeida-Toledo et al., 2000; [18]: Takagui et al., 2022.

**Table 2:** Samples of *Brachyhypopomus brevirostris* analyzed in this study.

Species	Locality	ID *	Sample
*Brachyhypopomus brevirostris*	Mamirauá Reserve—Tefé—AM/Amazon Basin	P-2635	1 ♀
	Aramanaí stream—Santarém—PA/Amazon Basin	P-3665; P-3667; P-3669	2♀/1 indetermined

Legend: (*) Ichthyology collection in the Centro de Estudos Avançados da Biodiversidade, CEABIO, UFPa, Brazil.

**Table 3:** Results of U2 snRNA sequences for Gymnotiformes available in the literature.

Specie (Localities)	2n	KF	snDNA U2	Reference
*Eigenmannia limbata* (Rio Branco—AC)	38	8m + 4sm + 26a	3 pairs (11, 12, 14)	[49]
*E. microstoma* (Francisco Dumont—MG)	38	8m + 10sm + 20a	4 pairs (10, 12, 16, 17)	[49]
*E.* aff. *trilineata* (Rio Miranda-Paraguai)	32	♂ 8m + 2sm + 22a ♀ 8m + 1sm + 22a	Simple (Par 12)	[50]
*Archolaemus janeae* (Altamira—PA and Santarém—PA)	46	4m/sm + 42st/a	3 pairs (3, 6, 13)	[51]
*Gymnotus pantanal* (Colômbia, SP)	40	4m + 3sm + 13st	7 pairs (9, 10, 11, 18, 20, X1)	[52]
*Gymnotus carapo* (Iquitos-Peru)	42	12m + 6sm + 3st	Simple (par 1)	[52]
*Gymnotus sylvius* (Botucatu, SP)	40	11m + 6sm + 3st	Simple (par 1)	[52]
*Gymnotus inaequilabiatus* (Botucatu, SP)	54	21m + 5sm + 1st	Simple (par 5)	[52]
*Gymnotus pantherinus* (Mongaguá, SP)	52	16m + 9sm + 1st	Simple (par 4)	[52]
*Gymnotus javari* (Iquitos-Peru)	50	6m + 4sm + 15st	Simple (par 11)	[52]

Legends: 2n = diploid number; KF = Karyotypic Formula; [49]: Araya-Jaime et al., 2022; [50]: Araya-Jaime et al., 2017; [51]: Rodrigues et al., 2021; [52]: Utsunomia et al., 2014.

## Data Availability

Data are contained within the article and Supplementary Materials.

## Supplementary Materials

The following supporting information can be downloaded at: https://www.mdpi.com/article/10.3390/ani14121726/s1, Table S1: Valid species comprising the genus *Brachyhypopomus*.
