# Supplementary material for: Mapping of Repetitive Sequences in Brachyhypopomus brevirostris (Hypopomidae, Gymnotiformes) from the Brazilian Amazon
Source: Animals (Basel). 2024 Jun 7;14(12):1726. doi: 10.3390/ani14121726 (PMC11200435; doi:10.3390/ani14121726)
Supplement: Supplementary file 1 [file animals-14-01726-s001.zip › animals-2955513-supplementary.pdf]

Supplementary Table S1 – Valid species comprising the genus *Brachyhypopomus*

**Genus** *Brachyhypopomus*	**Author, year**
B. brevirostris	Steindachner,1868
B. occidentalis	Regan, 1914
B. beebei	Schultz, 1944
B. diazi	Fernández-Yépez, 1972
B. pinnicaudatus	Hopkins, Comfort, Bastian & Bass, 1990
B. janeiroensis	Costa & Campos-da-Paz, 1992
B. jureiae	Triques & Khamis, 2003
B. bombilla	Loureiro & Silva, 2006
B. draco	Giora, Malabarba & Crampton, 2008
B. gauderio	Giora & Malabarba, 2009
B. bullocki	Sullivan & Hopkins, 2009
B. walteri	Sullivan, Zuanon & Fernandes, 2013
B. bennetti	Sullivan, Zuanon & Fernandes, 2013
B. alberti	Crampton, De Santana, Waddell, & Lovejoy, 2016b
B. arrayae	Crampton, De Santana, Waddell, & Lovejoy, 2016b
B. batesi	Crampton, De Santana, Waddell, & Lovejoy, 2016b
B. belindae	Crampton, De Santana, Waddell, & Lovejoy, 2016b
B. benjamini	Crampton, De Santana, Waddell, & Lovejoy, 2016b
B. cunia	Crampton, De Santana, Waddell, & Lovejoy, 2016b
B. flavipomus	Crampton, De Santana, Waddell, & Lovejoy, 2016b
B. hamiltoni	Crampton, De Santana, Waddell, & Lovejoy, 2016b
B. hendersoni	Crampton, De Santana, Waddell, & Lovejoy, 2016b
B. menezesi	Crampton, De Santana, Waddell, & Lovejoy, 2016b
B. palenque	Crampton, De Santana, Waddell, & Lovejoy, 2016b
B. provenzanoi	Crampton, De Santana, Waddell, & Lovejoy, 2016b
B. regani	Crampton, De Santana, Waddell, & Lovejoy, 2016b
B. sullivani	Crampton, De Santana, Waddell, & Lovejoy, 2016b
B. verdii	Crampton, De Santana, Waddell, & Lovejoy, 2016b
B. degy	Dutra et al. 2021
